# Identifying skills required of new epidemiologists: a content analysis of Canadian job postings and master’s programs

**DOI:** 10.3389/fpubh.2024.1418494

**Published:** 2024-09-19

**Authors:** Karli E. Chalmers, Kelsey L. Spence

**Affiliations:** Department of Population Medicine, Ontario Veterinary College, University of Guelph, Guelph, ON, Canada

**Keywords:** epidemiologist, epidemiology education, competencies, public health, curriculum development

## Abstract

**Introduction:**

The rise of emerging public health threats has increased the need for qualified epidemiologists in Canada. Our study aimed to identify the knowledge, skills, and abilities (KSAs) required of epidemiologists entering the workforce and determine whether these align with those taught in graduate epidemiology programs.

**Methods:**

An inductive content analysis of Canadian job postings from May to December 2023 containing the keyword “epidemiology” and requiring master’s degrees in epidemiology or related fields was conducted to identify the KSAs required in the workforce. Inductive content analysis of Master of Science (MSc) program descriptions and core course descriptions was completed to discern skills gained through Canadian graduate epidemiology and public health programs.

**Results:**

Based on the 295 job postings analyzed, five KSA categories were identified: communication skills (*n* = 268, 90.8%), analytical skills (*n* = 267, 90.5%), soft skills (*n* = 254, 86.1%), research methodology (*n* = 217, 73.6%), and knowledge of epidemiological concepts (*n* = 170, 57.6%). Analysis of 18 MSc programs found that that all of them described analytical skills, research methodology, and epidemiological concepts within their curriculum. Communication skills were described in 94.4% (*n* = 17) of programs, while soft skills were mentioned in 50.0% (*n* = 9). However, only 66.7% (*n* = 12) of programs outlined learning objectives or specified the skills acquired from their programs in their descriptions.

**Conclusion:**

There was alignment between the needs of the Canadian epidemiology job market and MSc programs, particularly in analytical skills and research methodology. However, development of soft skills should be emphasized within graduate epidemiology programs to better prepare graduates for the job market. Future research should aim to develop competency statements for epidemiologists in training to ensure consistency across graduate programs and promote career readiness.

## 1 Introduction

The COVID-19 pandemic highlighted the importance and many roles of individuals within the public health system (1). While those in frontline positions, such as nurses and physicians, focus on the treatment and prevention of disease, others, such as epidemiologists, often work behind the scenes to influence public health policy (2). Epidemiologists help inform the implementation of public health measures by identifying risk factors, analyzing the distribution, and assessing the frequency of diseases within populations (3). In addition to their role within public health systems, epidemiologists work in various other sectors and can specialize in areas such as infectious disease, environmental epidemiology, pharmacoepidemiology, and more (4).

In 2022, the Public Health Agency of Canada (PHAC) identified several emerging threats to the Canadian public health system, including climate change, antimicrobial resistance, the opioid crisis, and mental health challenges (5). As a result, there is a growing demand for epidemiologists in Canada, underscoring the need for individuals entering the workforce to be equipped with the competencies necessary to address these challenges (6). Competencies refer to the combination of knowledge, skills, and abilities (KSAs) expected from all individuals within a specific field (7, 8). Several core competencies for epidemiologists have previously been identified, primarily focusing on analytical and research skills including critical appraisal, data collection, analysis, and interpretation, and proficiency in at least one statistical software (9–13). However, historically, these core competencies have been developed outside of Canada (8–13). While the Association of Public Health Epidemiologists of Ontario developed core competencies for public health epidemiologists in 2007, these competencies were not widely distributed, and the advancement of technology and growing public health concerns necessitates an assessment of the KSAs required by epidemiologists today (7, 14).

In Canada, although epidemiology training can differ across specialties, the level of education across the field is relatively consistent – epidemiologists typically pursue a master’s degree in public health, epidemiology, population medicine, or other related fields (9, 15). Within this education requirement, students can choose from different degree programs, such as research-based or thesis-based Master of Science (MSc) programs, course-based MSc programs, or professional course-based Master of Public Health (MPH) programs (8, 9). Given the diversity of educational pathways for epidemiologists, it is crucial to assess whether these graduate programs adequately equip epidemiology students with the core competencies of the field.

Competency-based education is used in many disciplines to ensure students develop the KSAs required of their field (11). Professional course-based programs employ this framework through mandatory core courses that align with specific competencies; students then demonstrate their competencies through course deliverables (16). In 2008, PHAC developed Core Competencies for Public Health in Canada, consisting of 36 general KSAs organized into seven broader categories (17). These core competencies represent the competencies expected of all public health practitioners, including epidemiologists (17). To ensure a standardized skillset for MPH graduates across Canada, MPH programs must use the PHAC Core Competencies for curriculum development (16, 18). However, these competencies are not required by research-based (i.e., non-professional) MSc programs, leaving epidemiology program curricula at the discretion of each university. While there are only 19 MPH programs in Canada, there are more than 40 MSc programs related to epidemiology. As a result, the majority of Canadian epidemiologists may graduate from programs without a common set of competencies established among their students. Therefore, it is essential to identify the competencies taught in MSc in epidemiology programs in Canada and whether these align with KSAs required of epidemiologists entering the workforce.

The objectives of this study were to (a) identify the KSAs required of epidemiologists entering the Canadian job market, (b) evaluate the alignment of these KSAs with those gained from MSc in epidemiology and public health programs based on program and core course descriptions, and (c) appraise whether the PHAC Core Competencies Version 1.0 align with the KSAs identified through job postings.

## 2 Materials and methods

### 2.1 Job postings

#### 2.1.1 Search period and methodology

Job postings were collected from May 8th, 2023 to December 31st, 2023 across 12 online job boards including Indeed, Eluta, Monster, Glassdoor, LinkedIn, Workopolis, Charity Village, and a variety of province- and territory-specific government job portals. Within the job boards, the following Boolean search terms were applied: “epidemiology,” and (“epidemiology” + “masters”) to ensure that jobs contained the specified word(s) in the posting. Job postings that met the inclusion criteria were collected daily and duplicate postings across job boards were excluded from data collection. If positions were reposted, they were collected and marked accordingly, and then later excluded from analysis. Data were imported into NVivo Release 1.71 (QSR International Pty Ltd., London, UK) after being downloaded from online webpages using NCapture, a Google Chrome extension that allows web content to be uploaded into NVivo. Data were collected by one researcher (KC) using the same computer and browser.

#### 2.1.2 Inclusion and exclusion criteria

Inclusion criteria for job postings included: (a) jobs in Canada, (b) containing the keyword “epidemiology,” and (c) requiring or preferring a master’s degree.

As this study aimed to identify the skills required of epidemiologists entering the Canadian workforce (i.e., new graduates), we excluded jobs geared towards current students and those that required additional certifications and doctoral degrees. Exclusion criteria included: (a) student positions (i.e., summer students, co-op positions, Government of Canada Research Affiliate Program), (b) jobs requiring Certification in Infection Control (CIC), (c) jobs requiring doctoral and professional degrees, or certification/licensing by a governing body (i.e., PhD, DrPH, DVM, MD, RN), (d) job postings that did not specify education requirements and (e) jobs requiring significant leadership experience.

#### 2.1.3 Descriptive analysis

The following descriptive variables were collected from each job posting that met all inclusion criteria:

- Job Title: Job titles were collected, and positions were assigned to one of the following categories: (a) Researcher, (b) Analyst, (c) Epidemiologist, (d) Specialist, (e) Manager, (f) Program Lead, (g) Biostatistician, (h) Scientist, (i) Director, and (j) Other.
- Industry: All job postings were categorized by industry: (a) Government, (b) Academia, (c) Healthcare, (d) Non-Profit Organization, (e) Pharmaceutical Industry, and (f) Other, including consulting agencies and biotechnology industries.
- Location: The province or territory of each job posting was recorded. Jobs were assigned “Canada-wide” if the job posting specified that the role could be completed fully remotely and within any region of Canada.
- Education (Required/Preferred): Data were recorded for both required and preferred qualifications. Education requirements were classified as (a) Master’s Degree in Epidemiology, Public Health, or Related Field, (b) Master’s Degree (any), and (c) Master’s Degree Preferred.
- Work Experience: The number of years of experience required was collected and categorized as: (a) 1 to 2 years, (b) >2 to 3 years, (c) >3 to 4 years, (d) >5 years, and (e) Not Listed.

Descriptive characteristics were summarized using frequency distributions for categorical variables.

#### 2.1.4 Content analysis

Content analysis is a method used to descriptively analyze textual data and identify recurring themes by assigning codes (e.g., keywords or short phases) across the dataset (19). Content analysis can be inductive or deductive. In inductive content analysis, codes are generated directly from the textual data, whereas deductive content analysis applies previously determined codes to a new dataset (19). First, all data were read several times by KC to become familiar with the content before beginning the content analysis (20). An inductive line-by-line coding approach was used by KC, who assigned codes in the form of KSAs (e.g., knowledge of public health systems, oral communication, critical thinking) to describe the competencies outlined in the qualifications, requirements, and asset sections of job postings. After all job postings were coded, the codes were reviewed and sorted into broader KSA categories (e.g., analytical skills, communication skills, research methodology) (19). While only one author (KC) coded the data, consistent with the content analysis process described by Elo & Kyngas (19), the research team (KC and KS) continuously discussed the raw data, codes, and data interpretation to increase reliability of the results. Codes and their final KSA categories were developed and finalized through consensus between both authors.

Deductive content analysis was used to map the categories of the PHAC Core Competencies to the KSAs required in the job postings. The seven categories of PHAC Core Competencies include: Public Health Sciences; Assessment and Analysis; Policy and Program Planning, Implementation and Evaluation; Partnerships, Collaboration and Advocacy; Diversity and Inclusiveness; Communication; and Leadership (17). After initial familiarization of the data and the PHAC Core Competency descriptions, KC read each job posting and deductively applied codes aligning with these descriptions. For example, a job posting indicating “verbal communication skills” was deductively coded to the PHAC Core Competency category of “Communication Skills.” Similar to the inductive content analysis process described above, both authors (KC and KS) continuously discussed the raw data, codes, and data interpretation before reaching consensus on codes that should be applied. Diversity and Inclusiveness was not included in the analysis because it was absent in the majority of postings.

#### 2.1.5 Statistical analysis

Statistical analyses were conducted to explore whether industry, job titles, and level of education was associated with KSA requirements. Chi-square tests were used to identify the statistical significance of the results, which was set at *α* = 0.05. RStudio Version 2023.06.0 + 421 was used for statistical analyses.

### 2.2 MSc programs

#### 2.2.1 Search period and methodology

A list of Canadian universities offering MSc degrees related to epidemiology was generated using publicly available data from the Canadian Society for Epidemiology and Biostatistics website, and from a Google search for “MSc epidemiology programs in Canada” to ensure all programs were included (21). Programs were initially identified in May 2023, and core course descriptions were collected in October 2023; the search was conducted again on March 18th, 2024 to verify results.

#### 2.2.2 Inclusion and exclusion criteria

Inclusion criteria for programs included: (a) MSc programs in Canada, and (b) either thesis or course-based degrees focused on epidemiology, public health, or population medicine.

Exclusion criteria included: (a) MPH programs, and (b) MSc programs that only had optional specializations in epidemiology, public health, or population medicine.

#### 2.2.3 Content analysis

After applying exclusion criteria to the MSc programs, and thus only including programs that had mandatory specializations and/or focuses on epidemiology, public health, or population medicine, 18 MSc programs were included in this content analysis, which followed the same procedures as described above. Program descriptions, including any program objectives, were collected from university websites. These descriptions, along with names of all required core courses and calendar descriptions of each core course were imported into NVivo. In instances where students could choose between several course options to meet a requirement, all options were included. Solely optional or elective courses were excluded.

Inductive content analysis was used to identify the competencies acquired from each program, using both the program and core course descriptions. After all codes were identified, they were reviewed and arranged into the same broader KSA categories as the job postings (e.g., analytical skills, communication skills, research methodology, etc.) to determine the alignment between the needs of the job market and MSc program curricula (19).

## 3 Results

### 3.1 Job postings

#### 3.1.1 Descriptive analysis

A total of 295 unique job postings were included in the analysis (Table 1). The most common industry for job postings was government positions (*n* = 126, 42.7%), followed by healthcare (*n* = 56, 19.0%), and academia (*n* = 47, 15.9%). Nearly 56% (*n* = 165) of postings were for jobs located in Ontario. A variety of job titles was observed; 24.7% of job postings (*n* = 73) were for “researchers,” 20.3% (*n* = 60) were for “analysts,” and 20.0% (*n* = 59) were for “epidemiologists.” Most postings (*n* = 235, 79.7%) required a master’s degree, with 66.1% (*n* = 195) of total postings specifying a master’s degree in epidemiology, public health, or a related field. Nearly 27% (*n* = 79) of job postings specified the need for more than 2–3 years of experience, while a similar proportion required over 3–4 years of experience (*n* = 64, 21.7%). Approximately 21% (*n* = 63) of postings did not specify experience requirements.

**Table 1:** Descriptive characteristics of epidemiology job postings in Canada (*n* = 295) collected across online job boards from May to December 2023.

Variables	Number of job postings (%)
Industry	
Government	126 (42.7)
Healthcare	56 (19.0)
Academia	47 (15.9)
Non-profit	33 (11.2)
Other^1^	27 (9.15)
Pharmaceutical	6 (2.03)
Education requirement	
Master’s Degree in Epidemiology, Public Health, or Related	195 (66.1)
Master’s Degree Preferred	60 (20.3)
Master’s Degree (not specific)	40 (13.6)
Job title	
Researcher^2^	73 (24.7)
Analyst	60 (20.3)
Epidemiologist	59 (20.0)
Other^3^	23 (7.80)
Specialist	19 (6.44)
Manager	21 (7.12)
Program lead	17 (5.76)
(Bio)statistician	11 (3.73)
Scientist	8 (2.71)
Director	4 (1.36)
Experience required	
1–2 years	23 (7.80)
>2–3 years	79 (26.8)
>3–4 years	64 (21.7)
>4–5 years	13 (4.41)
>5 years	53 (18.0)
Not listed	63 (21.4)
Location	
Ontario	165 (55.9)
British Columbia	56 (19.0)
Alberta	34 (11.5)
Quebec	12 (4.07)
Nova Scotia	9 (3.05)
Manitoba	5 (1.69)
Saskatchewan	7 (2.37)
New Brunswick	1 (0.34)
Newfoundland and Labrador	2 (0.68)
Northwest Territories	2 (0.68)
Canada-Wide	1 (0.34)
Yukon	1 (0.34)

^1^Other options included: Consulting (*n* = 4), Biotechnology (*n* = 2), Research Companies (*n* = 12), Analytics (*n* = 2), among others. ^2^Researcher: Research Assistant (*n* = 17), Research Associate (*n* = 15), Research Coordinator/Manager/Advisor/Officer (*n* = 26), Research Methodologist (*n* = 9), Researcher (*n* = 6). ^3^Other options included: Advisor (*n* = 1), Business Development Representative (*n* = 1), Data Science and Evaluation Consultant (*n* = 1), Data Science and Evaluator Coordinator (*n* = 1), Health Economist (*n* = 3), Health Promoter (*n* = 1), Medical Writer (*n* = 2), Planning Officer (*n* = 1), among others.

#### 3.1.2 KSAs identified across job postings

Five categories of KSAs were identified: communication skills, analytical skills, soft skills, research methodology, and knowledge of epidemiological concepts (Table 2). Communication skills (*n* = 268, 90.8%) and analytical skills (*n* = 267, 90.5%) were most consistently required across job postings. The top three overall KSAs were partnership, collaboration, and teamwork (*n* = 198, 67.1%), written communication skills (*n* = 185, 62.7%), and data analysis (*n* = 167, 56.6%). A detailed list of all KSAs identified (i.e., the code book) can be found in Supplementary Table S1.

**Table 2:** Top five KSAs identified across epidemiology job postings in Canada (*n* = 295) collected across online job boards from May to December 2023, per KSA category.

Knowledge, skills, abilities (KSAs)	Number of job postings (%)
Communication skills	268 (90.8)
Written	185 (62.7)
Oral	162 (54.9)
Interpersonal skills	126 (42.7)
General communication skills^1^	90 (30.5)
Report development	88 (29.8)
Analytical skills	267 (90.5)
Data analysis	167 (56.6)
Statistical software	164 (55.6)
Statistical analysis	125 (42.4)
Data interpretation	94 (31.9)
General analytical skills^2^	89 (30.2)
Soft skills	254 (86.1)
Partnership, collaboration, and teamwork	198 (67.1)
Independent worker	125 (42.4)
Organization	118 (40.0)
Project management	110 (37.3)
Problem-solving	95 (32.2)
Research methodology	217 (73.6)
Research methods	96 (32.5)
Quantitative methods	84 (28.5)
Research experience	75 (25.4)
Qualitative methods	52 (17.6)
Study design	45 (15.3)
Knowledge of epidemiological concepts	170 (57.6)
Principles of epidemiology and experience with concepts^3^	68 (23.1)
Public health knowledge and experience	57 (19.3)
Provincial health systems	39 (13.2)
Surveillance	30 (10.2)
Communicable disease	23 (7.80)

^1General communication skills was coded for postings that did not specify oral or written communication skills. 2General analytical skills was coded for postings that included “analytical skills” as a requirement.3Principles of epidemiology was coded in instances where job postings indicated that experience applying epidemiology concepts was required but did not specify particular epidemiological measurements.^

#### 3.1.3 KSA categories by industry

**Figure 1:**
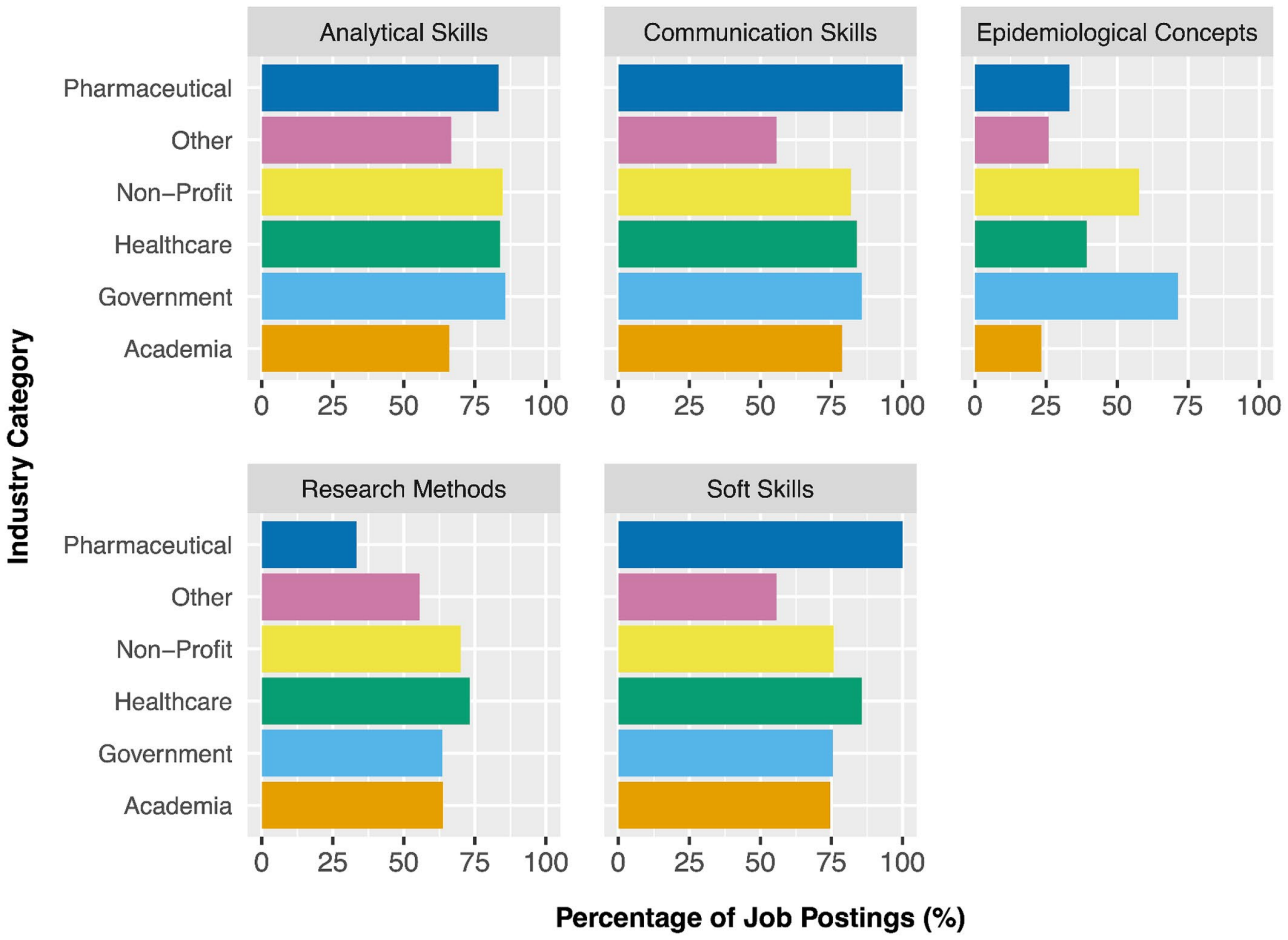
Comparison of knowledge, skills, and attributes (KSA) categories required across each industry, collected from online job postings across Canada (*n* = 295).

Figure 1 demonstrates the percentage of job postings within each industry that require skills from each KSA category. The distribution of analytical skills required among each of the industries was relatively even, with these skills being required of 85.7% of government postings (*n* = 108), 83.9% of healthcare (*n* = 47), 84.8% of non-profit (*n* = 28), and 83.3% of pharmaceutical postings (*n* = 5). Communication skills were required of 100% of pharmaceutical industry positions (*n* = 6), 85.7% (*n* = 108) of government postings, 83.9% (*n* = 47) of healthcare postings, and 78.7% (*n* = 37) of academia postings. Similarly, the percentage of postings requiring research methods and soft skills were comparable across industry. Postings within the healthcare sector had the highest demand for skills and knowledge pertaining to research methods (*n* = 41, 73.2%), as well as soft skills (*n* = 48, 85.7%). Of all KSA categories, knowledge of epidemiological concepts was one of the least mentioned (*n* = 170, 57.6%). Government positions showed the highest demand for these skills at 71.4% (*n* = 90), followed by the non-profit industry where 57.6% (*n* = 19) of postings required skills within this category.

#### 3.1.4 KSA categories by position title

Knowledge of epidemiological concepts were significantly in higher demand among positions titled “epidemiologist,” with 91.5% (*n* = 54) of these jobs requiring expertise in at least one epidemiological concept (χ^2^ = 48.0304, *p* < 0.05). Knowledge of epidemiological concepts were also significantly higher among job postings that required a master’s degree in epidemiology, public health, or a related field (χ^2^ = 6.383, *p* < 0.05) compared to job postings not requiring specific master’s degrees, or those considering master’s degrees to be an asset. Soft skills were required in all positions, with an emphasis on directors (*n* = 4, 100%), managers (*n* = 21, 100%), program leads (*n* = 15, 88.2%), and specialists (*n* = 16, 84.2%).

Of the 170 job postings that included knowledge and experience pertaining to epidemiological concepts, only two job postings specifically indicated which concepts were required, including knowledge of odds ratios, risk ratios, confidence intervals, and sample size calculations. The majority of postings requiring epidemiology knowledge included broad statements, such as “Demonstrated ability to apply theories, concepts, methods and measures in epidemiology.”

#### 3.1.5 Software proficiencies

A total of 214 (72.5%) of postings indicated the requirement of proficiency in at least one computer software (Table 3). Nearly half of the job postings (*n* = 144, 48.8%). requested experience with MS Office, including Word, Excel, and PowerPoint. Proficiency in at least one statistical software was required among 50.5% of postings (*n* = 149). The statistical software R was most highly requested in academia job postings (*n* = 15, 31.9%), whereas SAS was the most common statistical software included in government postings (*n* = 42, 33.3%). Proficiency in Excel was most highly requested in the pharmaceutical industry, with two-thirds of job postings requiring knowledge of this software (*n* = 4, 66.7%). STATA and SPSS were the least sought after statistical software across industries. A breakdown of how statistical software requirements varied by industry can be found in Supplementary Figure S1.

**Table 3:** Software requirements identified across epidemiology job postings in Canada (*n* = 295) collected across online job boards from May to December 2023.

Software program^1^	Number of job postings (%)
Microsoft Office, including Excel	144 (48.8)
SAS	105 (35.6)
R/RStudio	99 (33.6)
SPSS	45 (15.3)
STATA	48 (16.3)
SQL	38 (12.9)
Python	37 (12.5)
Microsoft Access	25 (8.47)
PowerBI	19 (6.44)
Tableau	17 (5.76)
NVivo	14 (4.75)
GIS Software	12 (4.07)
EpiInfo	6 (2.03)
Epi Data	1 (0.34)

^1Software requirements are not unique to any individual postings. Several postings indicated proficiency in more than one software.^

#### 3.1.6 Comparison with PHAC core competencies

Deductive content analysis revealed that 281 postings (95.3%) required KSAs aligning with those outlined in the PHAC Assessment and Analysis category, while 268 postings (90.8%) aligned with the Communication competencies. Leadership competencies were required among 241 (81.7%) of jobs. Partnerships, Collaboration, and Advocacy were required of 198 jobs (67.1%), while Public Health Sciences was required of 160 jobs (54.2%). Policy and Program Planning, Implementation, and Evaluation were required of only 64 jobs (21.7%).

### 3.2 Master’s programs

#### 3.2.1 Program and core course descriptions

A total of 18 programs were included in the analysis (Supplementary Table S2). Of these programs, only 12 (66.7%) had publicly available descriptions that clearly outlined program objectives and/or KSAs acquired by their students.

Within the program objectives and general descriptions, 55.6% of programs (*n* = 10) mentioned the development of analytical skills, 61.1% (*n* = 11) included knowledge of epidemiological concepts, and 72.2% (*n* = 13) included skills and knowledge pertaining to research methodology. Only half of the programs (*n* = 9, 50.0%) indicated the development of communication skills in their descriptions, whereas one-third (*n* = 6, 33.3%) referenced development of soft skills. The top skills emphasized in program descriptions were study design (*n* = 9, 50.0%), research methods (*n* = 7, 38.9%), and critical appraisal (*n* = 7, 38.9%).

Inductive coding of all core course descriptions revealed that all programs (*n* = 18, 100%) required students to take courses that emphasized development of analytical skills, research methodology, and knowledge of epidemiological concepts. However, only 66.7% of programs (*n* = 12) offered courses that outlined the development of communication skills, while one-third (*n* = 6, 33.3%) included the development of soft skills in their core course descriptions. The top three skills identified in course descriptions included knowledge of epidemiological concepts (*n* = 18, 100%), study design (*n* = 15, 83.3%), and data analysis (*n* = 15, 83.3%).

#### 3.2.2 Overall KSAs obtained by MSc programs

Among both the MSc program descriptions and core course descriptions, 100% of programs (*n* = 18) included the development of analytical skills, research methodology, and knowledge of epidemiological concepts (Table 4). The top three skills coded in MSc programs overall include study design (*n* = 18, 100%), knowledge of epidemiological concepts (*n* = 18, 100%), and data analysis (*n* = 16, 88.9%). Nearly all programs (*n* = 17, 94.4%) included the development of communication skills; however, this was mainly focused on report writing only. Half of the programs (*n* = 9, 50.0%) mentioned soft skills in either the program description or core course descriptions. Only four programs (22.2%) described skills pertaining to partnership and collaboration.

**Table 4:** Top five KSAs identified in program and core course descriptions of Canadian MSc in epidemiology and public health programs, per KSA category.

Knowledge, skills, abilities (KSAs)	Number of MSc programs (%)
Communication skills	17 (94.4)
Research report development	11 (61.1)
Presentation skills	8 (44.4)
Written	3 (16.7)
Knowledge translation	2 (11.1)
Oral	2 (11.1)
Analytical skills	18 (100)
Data analysis	16 (88.9)
Statistical analyses	15 (83.3)
Data interpretation	9 (50.0)
Statistical software	8 (44.4)
Data collection	8 (44.4)
General analytical skills^1^	8 (44.4)
Soft skills	9 (50.0)
Partnership, collaboration, and teamwork	5 (27.8)
Critical thinking	3 (16.7)
Project management	1 (5.56)
Problem-solving	1 (5.56)
Time management	1 (5.56)
Knowledge of research methods	18 (100)
Study design	17 (94.4)
Research methodology	12 (66.7)
Quantitative methods	11 (61.1)
Qualitative methods	9 (50.0)
Research ethics	8 (44.4)
Knowledge of epidemiological concepts	18 (100)
Principles of epidemiology	18 (100)
Knowledge of epidemiologic studies including observational studies	13 (72.2)
Critical appraisal	12 (66.7)
Sources of bias and confounding	10 (55.6)
Determinants of health	7 (38.9)

^1General analytical skills was coded for descriptions that included “analytical skills” as a skill acquired.^

## 4 Discussion

The objectives of this study were to identify the KSAs required of epidemiologists within the Canadian workforce and determine if they aligned with those taught in Canadian MSc in epidemiology or public health programs, as well as with the PHAC Core Competencies for Public Health in Canada (17). Five KSA categories were identified through the postings, including communication skills, analytical skills, knowledge of research methods, soft skills, and knowledge of epidemiological concepts. These KSAs were well-aligned with the PHAC Core Competency categories of Assessment and Analysis, Communication, Partnership and Collaboration, Leadership, and Public Health Sciences, indicating that these previously established competencies for MPH programs may be appropriate for curriculum development among MSc programs (17). Based on program and core course descriptions, Canadian MSc in epidemiology programs are aligned with the current needs of the job market. However, this alignment is notably stronger for analytical skills, research methodology, and knowledge of epidemiological concepts, compared to communication and soft skills.

The distribution of KSA categories were generally consistent across job postings in different industries. Most job postings within each industry, and nearly 91% of job postings across the dataset, required communication skills, primarily pertaining to written communication, oral communication, and interpersonal skills. As demonstrated through the COVID-19 pandemic, communication skills are of utmost importance for epidemiologists (22). As epidemiologists develop an understanding of disease determinants and distribution, they must be able to effectively explain this information to policymakers and members of the public (22). While almost all MSc programs included in this analysis addressed communication skills in their program or core course descriptions, the emphasis was largely on research report writing. Therefore, MSc programs can better prepare graduates through increasing opportunities for oral communication and written assignments tailored for public audiences, such as public health releases.

While communication and analytical skills were the most common KSA category included in the job postings, the single most consistently requested skill among all job postings was those pertaining to partnership and collaboration, appearing in 67.1% of postings. This aligns with previous findings and represents a potential newer trend and skill requirement among epidemiologists (7). As there is an increasing need to include members of the public, healthcare providers, and policymakers in health decision-making, it is essential that epidemiologists have the skillset necessary to effectively engage with these partners and identify appropriate collaborators (7). Furthermore, as approaches to improve trans-disciplinarity among epidemiologists are increasing, partnership and collaboration skills are necessary for appropriately engaging with other disciplines (23). Additional learning opportunities within graduate programs should be added to enhance skill building in this area.

Analytical skills, particularly skills in data analysis, data interpretation, and use of statistical software, were required by the majority of postings for each industry and over 90% of postings overall. These skills are consistent with previous studies that indicated that skills in data collection, data analysis, data interpretation, and proficiencies in statistical software are core competencies for epidemiologists (7–10, 24). As this observation aligns closely with previous research, graduate programs should continue to emphasize these analytical skills in their curricula. Interestingly, only 65.6% (*n* = 31) of jobs within academia mentioned at least one analytical skill in their job postings. Many of the postings that did not include analytical skills – both in academia and other industries – were for research assistants that either did not require master’s degrees, or required minimal work experience (i.e., minimum 1–2 years). Thus, these positions may be most suitable for new epidemiology graduates who are looking to gain experience.

As analytical skills were included in many of the MSc program and core course descriptions and will be acquired through any research projects associated with these programs, graduates may be well-prepared for this KSA requirement. The same conclusion can be made regarding the requirement of knowledge of research methodology. However, special attention should be paid to the software most currently in-demand by employers. Little information was publicly available pertaining to the statistical software that most MSc programs use; however, instructors may be encouraged to emphasize the use of SAS or R/RStudio given the demands in the workforce.

While soft skills were included in approximately 86% of job postings, only half of the MSc programs included in this study referenced these skills in their program or core course descriptions. This finding may be limited by the lack of publicly available information on the skills gained by MSc programs. At the outset of this research, we had planned to analyze the learning outcomes (LOs) from each MSc program to identify whether the KSAs required by employers are included in existing curricula. However, we found that many programs did not have publicly available LOs, necessitating the use of program and core course descriptions to discern skills acquired from graduate epidemiology programs. As soft skills are often considered professional skills, typically developed through experience rather than formal class instruction, it is possible that graduate students are receiving training on these skills outside of class time (25). However, none of the programs included in this analysis had practicums or practice-based learning experiences as degree requirements. Given the demand of these skills in the job market, it is clear that employers require well-rounded candidates. Thus, graduate programs are encouraged to develop methods to improve education in this area.

Within the job postings, most variation in KSAs was observed among the requirement of knowledge of epidemiological concepts. While the majority of government positions required candidates to have experience applying epidemiological concepts, this was not observed for any other industry. However, the language used to describe this KSA category was very broad, and only two postings specifically listed examples of epidemiological concepts (i.e., sample size calculations, odds, and risk ratios). Thus, it remains unclear what constitutes these requirements and highlights a potential lack of transparency in job postings, making it difficult to identify if MSc in epidemiology programs are adequately preparing graduates in this subject area. Alternatively, this may indicate that industries are willing to hire new graduates with limited experience applying epidemiological concepts as their master’s degree is indicative of their ability to learn these skills on the job. Interestingly, there was an association observed between jobs tilted “epidemiologist” and those requiring master’s degrees in epidemiology, public health, or a related field. These roles are more likely to involve tasks such as disease surveillance, reporting of public health issues, and public health and risk assessment compared to positions titled “researchers” or “analysts.” While this correlation provides insights into the specific skills needed for epidemiologists, we were not able to determine whether graduate epidemiology programs adequately provide the skills required by epidemiologists in this KSA category.

### 4.1 Limitations

The results of this study may be limited by the use of publicly available information. Job postings were collected from online job boards between May and December 2023, thus the job postings analyzed in this article may not be representative of jobs obtained through direct hires, or postings available only on internal sites. As most of the data for MSc programs was obtained from core course descriptions, there is a possibility that the identified skills acquired from these programs may be inaccurate if these descriptions are not regularly updated or fail to accurately reflect the content or structure of the course. Similarly, it is possible that MSc programs have developed LOs or program descriptions outlining the KSAs expected to be obtained by their graduates that are not publicly available on their website. Additionally, because all program-related data was collected from program websites, it is possible that information highlighted on the website does not accurately reflect the full content delivered in each program. To limit this bias, efforts were made to contact each MSc program’s primary contact during the planning stage of this study; however, few responses were received. Further work in this area is warranted to more fully understand the depth of each program’s curriculum and how it may align with the KSA categories reported in this study.

While there are more than 40 MSc programs relating to epidemiology in Canada, only 18 MSc programs were included in the analysis. As this study only included programs with mandatory specializations or areas of emphasis in epidemiology, public health, or population medicine, it is possible that if more programs were included in this analysis, the distribution of KSAs identified through the MSc programs may have differed. For example, there is a possibility that if other programs were included (i.e., those with optional epidemiology specializations and more interdisciplinary in nature), the proportion of MSc programs emphasizing the development of communication and/or soft skills may have increased. However, for the purposes of this study, we aimed to focus solely on epidemiology-specific programs and included all retrieved programs that met this inclusion criteria. Furthermore, although only a portion of MSc programs were included in this analysis, we included programs from nine of ten provinces within Canada, ensuring the sample was representative of a variety of geographic locations. However, because many of the universities were located within mid-to-large urban centers, it is possible that programs offered in more rural settings may offer different learning experiences to enhance skillsets.

Further, the job postings analyzed in this article represent the needs of the job market at one specific time. As technology advances, or as new public health threats emerge, the needs of Canadian epidemiologists may change. To address this limitation, this study collected data for 7 months. Throughout this duration, there was consistency in the requirements across sectors, with no discernible trends emerging over time. Because these job postings were collected during the COVID-19 pandemic, and some job postings were directly related to COVID-19 surveillance, this may have influenced the number of government postings and demand for new hires.

Lastly, only one member of the research team (KC) primarily coded the data. Given that content analyses and coding rely on the interpretation of textual data to collect data, it is possible that if another research team were to analyze the job postings and MSc program and core course descriptions, results (e.g., the KSA categories) may have differed. However, considering the nature of this content analysis and the goal of identifying the KSAs outlined in the texts, KC developed descriptive codes such as “oral communication” and “data analysis” to accurately represent these requirements as they appeared in the job postings and MSc program descriptions. Throughout the coding process, both authors (KC and KS) iteratively discussed the codes and data interpretation to increase reliability of the results, resolving any potential discrepancies or errors in coding. After the initial coding was complete, the authors discussed common skillsets and themes that emerged from the data, and consequently developed the KSA categories.

### 4.2 Future work and directions

This study was a required first step in identifying the KSAs required of Canadian epidemiologists. However, due to the broad language used in job postings, we were not able to discern the epidemiology-specific knowledge that is required of epidemiologists entering the workforce. Future work should involve epidemiologists and experts in the field to (a) identify the epidemiological concepts that epidemiologists regularly apply in their roles, and (b) ultimately develop competency statements that can be used to develop a curriculum framework for MSc in epidemiology programs. As the distribution of KSAs varied among industry (Figure 1), it would be beneficial to include epidemiologists across various industries to develop these competency statements. For example, while analytical skills were included in most postings for each industry, the knowledge of epidemiological concepts was most highly requested in government postings. Thus, developing industry-specific competencies in addition to overarching core competencies may help MSc programs and students tailor their learning to the needs of their desired industry.

We found that many Canadian MSc in epidemiology programs did not have publicly available LOs, making it difficult to assess whether the programs were rooted in competency-based education. As competency-based education has been found to improve student learning and readiness for the workforce, it is essential that MSc programs develop specific and measurable LOs (26). The results from this study can provide a framework (e.g., five KSA categories) for MSc programs to create LOs to enhance curricula and ensure the adequate preparation of epidemiologists for the workforce. Further, this study has identified a gap in MSc programs in terms of the development of soft skills. Thus, we encourage graduate epidemiology programs to explore avenues to increase the development and promotion of these skills unto their students or to identify whether they are being met outside of course curricula. For example, further exploration of the skills gained from research projects or theses completed as part of the MSc program would be highly beneficial. An outcome assessment of the University of Guelph’s Master of Public Health program, which requires a practicum of 12–16 weeks, found that most students said that the practicum was valuable and meaningful in gaining experience in the public health sector, dealing with challenges, and applying concepts learned through coursework (27). By incorporating a similar evaluation and reflection component into MSc programs, students will have the opportunity to self-assess skills gained through research and explore avenues for improvement.

This study lays the groundwork for future studies to investigate graduate epidemiology programs further. For example, while this study analyzed data from core course descriptions, a content analysis of course outlines may provide a more comprehensive understanding of the KSAs acquired from these programs. Additional work should investigate how graduate programs can be adapted to better prepare epidemiologists for the workforce.

## 5 Conclusion

This was the first study, to the research team’s knowledge, that analyzed both Canadian job postings and the program and core course descriptions of MSc in epidemiology and public health programs. With emerging public health threats, there is an increased need for qualified epidemiologists entering the workforce. From analyzing 295 unique job postings, we identified five KSA categories for Canadian epidemiologists, including communication skills, analytical skills, soft skills, knowledge pertaining to research methods, and knowledge of epidemiological concepts. These competencies align with several of the PHAC Core Competency categories, indicating that they may be suitable for curriculum development for MSc in epidemiology programs. Upon analyzing the program and core course descriptions for 18 MSc in epidemiology and public health programs in Canada, we discovered a general alignment between the KSAs acquired through these programs and the requirements of the job market. However, we identified a gap in education regarding the development of soft skills. Notably, we found that very few programs had publicly available LOs or thoroughly described the KSAs intended to be obtained by their students. This exacerbates the existing gap in graduate epidemiology education due to the absence of standardized competencies for MSc programs, in contrast to MPH programs. Given the diverse skillsets required of epidemiologists, graduate programs should ensure that their curricula encompass aspects of each of the five identified KSA categories. To equip epidemiologists with the necessary skillset to address escalating public health concerns, MSc programs should ensure they have specific and measurable LOs. Going forward, with the increasing demand for epidemiologists, it is imperative to develop core competencies tailored specifically for this field.

## Data Availability

The original contributions presented in the study are included in the article/Supplementary material, further inquiries can be directed to the corresponding author.
