# Supplementary material for: Identifying skills required of new epidemiologists: a content analysis of Canadian job postings and master’s programs
Source: Front Public Health. 2024 Sep 19;12:1418494. doi: 10.3389/fpubh.2024.1418494 (PMC11446749; doi:10.3389/fpubh.2024.1418494)
Supplement: Supplementary file 1 [file Data_Sheet_1.docx]

Supplementary Material

# Supplementary Data

**Supplementary Table 1.** All knowledge, skills, and abilities (KSAs) identified across epidemiology job postings in Canada (n=295) collected across online job boards from May to December 2023.

| **Knowledge, Skills, Abilities (KSAs)** | **Number of Job Postings** |
| --- | --- |
| **Communication Skills** | **268** |
| Written | 185 |
| Oral | 162 |
| Interpersonal Skills | 126 |
| General Communication Skills^1^ | 90 |
| Report Development | 88 |
| Presentation Skills | 67 |
| Knowledge Translation | 54 |
| Scientific Writing | 15 |
| Technical Writing | 10 |
| **Analytical Skills** | **267** |
| Data Analysis | 167 |
| Statistical Software | 164 |
| Statistical Analysis | 125 |
| Data Interpretation | 94 |
| General Analytical Skills^2^ | 89 |
| Data Management | 72 |
| Data collection | 60 |
| Data Visualization and Modeling | 49 |
| Administrative Databases [Health] | 53 |
| Big Data | 39 |
| Database Development and Management | 32 |
| Synthesize Info | 27 |
| Biostatistics Knowledge & Experience | 19 |
| Data Cleaning | 11 |
| **Soft Skills** | **254** |
| Partnership, Collaboration, and Teamwork | 198 |
| Independent Worker | 125 |
| Organization | 118 |
| Project Management | 110 |
| Problem-Solving | 95 |
| Prioritization | 101 |
| Time Management | 87 |
| Attention To Detail | 80 |
| Leadership | 85 |
| Initiative | 45 |
| Critical Thinking | 30 |
| **Research Methodology** | **218** |
| Research Methods | 96 |
| Quantitative Skills | 84 |
| Research Experience | 75 |
| Qualitative Research | 52 |
| Study Design | 45 |
| Publication | 36 |
| Literature Reviews | 25 |
| Systematic Review or Meta Analysis | 23 |
| Human Ethics Research | 18 |
| Survey Development | 16 |
| Reference Managers | 16 |
| Interviewing | 4 |
| Meta Analysis Software | 5 |
| Research Question Development | 4 |
| Scientific Journal Search Engines | 5 |
| GRADE | 3 |
| Covidence | 2 |
| **Knowledge of Epidemiological Concepts** | **172** |
| Principles of Epidemiology & Experience with Concepts^3^ | 68 |
| Public Health Knowledge & Experience | 57 |
| Provincial Health Systems | 39 |
| Surveillance | 30 |
| Canadian Health System | 22 |
| Communicable Disease | 23 |
| Trend Analysis and Forecasting | 20 |
| Critical Appraisal | 17 |
| [Social] Determinants of Health Knowledge | 19 |
| Community Health Knowledge & Experience | 18 |
| Mapping and Geospatial Analysis | 20 |
| Epidemiological Studies | 19 |
| Infectious Disease Epi | 17 |
| Public Health Data Systems Knowledge | 14 |
| Outbreak | 6 |
| Risk Assessment | 3 |
| Toronto's Health | 4 |
| Epi Math | 3 |
| Bias Sources | 2 |
| **Software Proficiencies** | **214** |
| MS Office | 144 |
| Excel | 104 |
| SAS | 105 |
| R | 99 |
| SPSS | 45 |
| STATA | 48 |
| SQL | 38 |
| Python | 37 |
| Microsoft Access | 25 |
| PowerBI | 19 |
| Tableau | 17 |
| NVivo | 14 |
| QGIS or ArcGIS | 12 |
| EpiInfo | 6 |
| Epi Data | 1 |
| **PHAC Core Competencies** | **295** |
| Assessment and Analysis | 281 |
| Communication | 268 |
| Leadership | 241 |
| Partnerships, Collaboration, and Advocacy | 198 |
| Public Health Sciences | 160 |
| Policy and Program Planning, Implementation and Evaluation | 64 |
| **Additional Unassigned KSAs** |  |
| Computer Proficiency | 130 |
| Facilitation | 40 |
| Privacy and Confidentiality | 28 |
| Planning | 31 |
| Policy Development | 27 |
| Programming Languages | 27 |
| Indigenous Health | 28 |
| Decision Making | 23 |
| Evaluation Methodology | 23 |
| Program Evaluation | 22 |
| Grant Writing | 19 |
| Performance Measurement | 16 |
| Program Implementation | 17 |
| Quality Improvement | 14 |
| Health Policy Knowledge | 13 |
| Public Health Reporting Systems | 12 |
| Clinical Trials | 12 |
| Cancer Data | 11 |
| Quality Assurance | 9 |
| Health Data Sources | 7 |
| IPAC Knowledge | 6 |
| Knowledge Synthesis | 6 |
| Special Populations | 6 |
| Dashboard Development | 3 |
| Data Programming | 4 |
| Machine Learning Algorithms | 5 |
| Policy Briefs | 5 |
| Relational Databases | 2 |
| Strategic Planning | 5 |
| Substance Abuse | 4 |
| Bibliographic Databases | 3 |
| Health Knowledge | 4 |
| Integrity | 2 |
| Listening Skills | 2 |
| Mental Health | 3 |
| Predictive Data Models | 4 |
| Teaching Skills | 4 |
| Chronic Disease Knowledge | 3 |
| HIV and Substance Abuse | 2 |
| Immunization | 3 |
| Indigenous Data | 3 |
| Innovation | 1 |
| Patient Centered Care | 3 |
| Pharmacoepidemiology | 3 |
| Text or Data Mining | 3 |
| Align to Organization | 2 |
| Child Health | 2 |
| Blood Borne Infections | 1 |
| Budget Management | 1 |
| Consulting | 1 |
| Curriculum Design | 1 |
| Environmental Health Knowledge | 1 |
| Health Records | 1 |
| Health Status Assessment | 1 |
| Health System Performance Measurement | 1 |
| Hospital Epidemiology | 1 |
| Indigenous Culture Knowledge | 1 |
| Infectious Disease Data Sources | 1 |
| Make Investigational Plan | 1 |
| Microbiology Lab Operations | 1 |
| Microbiology Lab | 1 |
| Sexual Health | 1 |
| Wastewater | 1 |

^1^General communication skills was coded for postings that did not specify oral or written communication skills. ^2^General analytical skills was coded for postings that included “analytical skills” as a requirement.^3^Principles of epidemiology was coded in instances where job postings indicated that experience applying epidemiology concepts was required but did not specify epidemiological measurements.

**
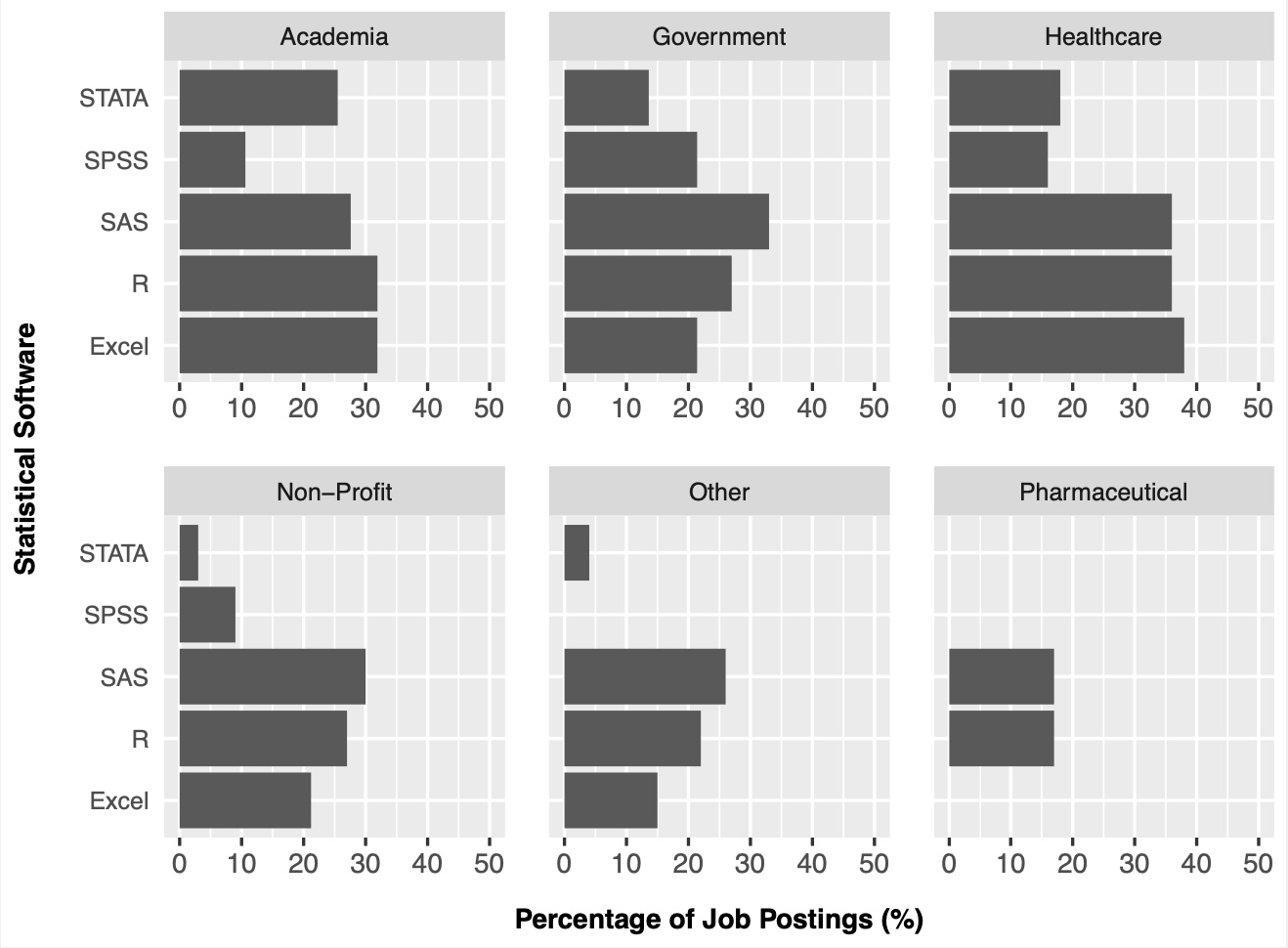
**

**Supplementary Figure 1.** Statistical software requirements across industry, demonstrated through percentage of job postings per industry.

**Supplementary Table 2.** List of Canadian MSc in Epidemiology or Public Health Programs included in Content Analysis

| **Province** | **School** | **Program & *Field (mandatory)*** |
| --- | --- | --- |
| Ontario | Queens University | MSc in Epidemiology |
|  | University of Guelph | MSc in Population Medicine  *Population Medicine (thesis-based)*  *Epidemiology (course-based)*  *Public Health (course-based)* |
|  | University of Ottawa | MSc in Epidemiology |
|  | University of Waterloo | MSc in Public Health Sciences |
|  | Western University | MSc in Epidemiology |
| Quebec | McGill University | MSc in Epidemiology |
|  | University of Montreal | MSc in Epidemiology |
|  | University of Laval | MSc in Epidemiology |
|  |  | MSc in Public Health |
| British Columbia | University of British Columbia | MSc in Population and Public Health |
| Alberta | University of Alberta | MSc in Public Health  *Epidemiology*  *General Public Health* |
|  | University of Calgary | MSc in Community Health Sciences  *Epidemiology*  *Population/Public Health* |
| Manitoba | University of Manitoba | MSc in Community Health Services |
| Saskatchewan | University of Saskatchewan | MSc in Community and Population Health Sciences |
| Nova Scotia | Dalhousie University | MSc in Epidemiology and Applied Health Research |
| Newfoundland and Labrador | Memorial University | MSc in Clinical Epidemiology |
|  |  | MSc in Community Health |
| Prince Edward Island | University of Prince Edward Island | MSc in Epidemiology |
